# In Silico Demonstration of Fast Anhydrous Proton Conduction on Graphanol

**DOI:** 10.1021/acsami.3c04022

**Published:** 2023-05-16

**Authors:** Siddarth K. Achar, Leonardo Bernasconi, Ruby I. DeMaio, Katlyn R. Howard, J. Karl Johnson

**Affiliations:** † Computational Modeling & Simulation Program, University of Pittsburgh, Pittsburgh, Pennsylvania 15260, United States; ‡ Department of Chemical & Petroleum Engineering, University of Pittsburgh, Pittsburgh, Pennsylvania 15261, United States; ¶ Center for Research Computing and Department of Chemistry, University of Pittsburgh, Pittsburgh, Pennsylvania 15260, United States

**Keywords:** proton conductivity, 2-D materials, density functional theory, machine learning, molecular dynamics, lattice Monte Carlo

## Abstract

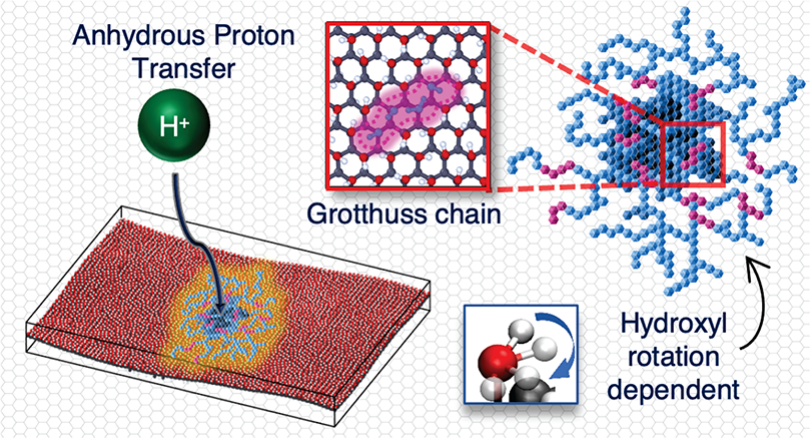

Development of new materials capable of conducting protons in the absence of water is crucial for improving the performance, reducing the cost, and extending the operating conditions for proton exchange membrane fuel cells. We present detailed atomistic simulations showing that graphanol (hydroxylated graphane) will conduct protons anhydrously with very low diffusion barriers. We developed a deep learning potential (DP) for graphanol that has near-density functional theory accuracy but requires a very small fraction of the computational cost. We used our DP to calculate proton self-diffusion coefficients as a function of temperature, to estimate the overall barrier to proton diffusion, and to characterize the impact of thermal fluctuations as a function of system size. We propose and test a detailed mechanism for proton conduction on the surface of graphanol. We show that protons can rapidly hop along Grotthuss chains containing several hydroxyl groups aligned such that hydrogen bonds allow for conduction of protons forward and backward along the chain without hydroxyl group rotation. Long-range proton transport only takes place as new Grotthuss chains are formed by rotation of one or more hydroxyl groups in the chain. Thus, the overall diffusion barrier consists of a convolution of the intrinsic proton hopping barrier and the intrinsic hydroxyl rotation barrier. Our results provide a set of design rules for developing new anhydrous proton conducting membranes with even lower diffusion barriers.

## 1 Introduction

Proton transport (PT) plays a vital role in many different fields. In this work we focus on the importance of PT in proton exchange membrane (PEM) hydrogen fuel cells. Current PEM fuel cells most commonly employ Nafion,^1^ a sulfonated fluoropolymer, as the PEM material. While this material is widely used and well-characterized, there are still questions about the precise mechanism and limitations of PT in hydrated Nafion. Because of this, there have been many recent studies using molecular simulations aimed at elucidating the atomistic details of the PT mechanism in Nafion and related materials under hydrated conditions.^2−8^

A problem with Nafion is that it is not an intrinsic proton conduction material and must be hydrated. This limits the operating temperature to <80 °C and requires complex and costly water management systems to maintain proper hydration without flooding.^9−12^ Developing membranes that can operate at elevated temperatures and at low humidity or under anhydrous conditions can enable increased kinetics, higher resistance to electrode poisoning, simplified water management, and reduced cost.^13−26^

Recent work on anhydrous or low humidity PEM materials involves metal–organic frameworks, covalent organic frameworks, coordination polymer materials, proton conducting polymers, ionic liquids, and other materials.^25−42^ These materials have not yet found use in practical PEM fuel cells, and each of them has their own unique challenges. There is thus a need to evaluate new materials for anhydrous or near-anhydrous PT. In silico methods are needed that involve predictive modeling of membranes at the atomic level, such as density functional theory (DFT), since they enable evaluation and screening of materials without expensive experimental efforts. However, methods such as DFT can only model small system sizes for short time scales because of computational limitations. This calls for simulation methods that can go beyond the lengths and time scales that DFT offers, while maintaining the accuracy that DFT provides.

**Figure 1:**
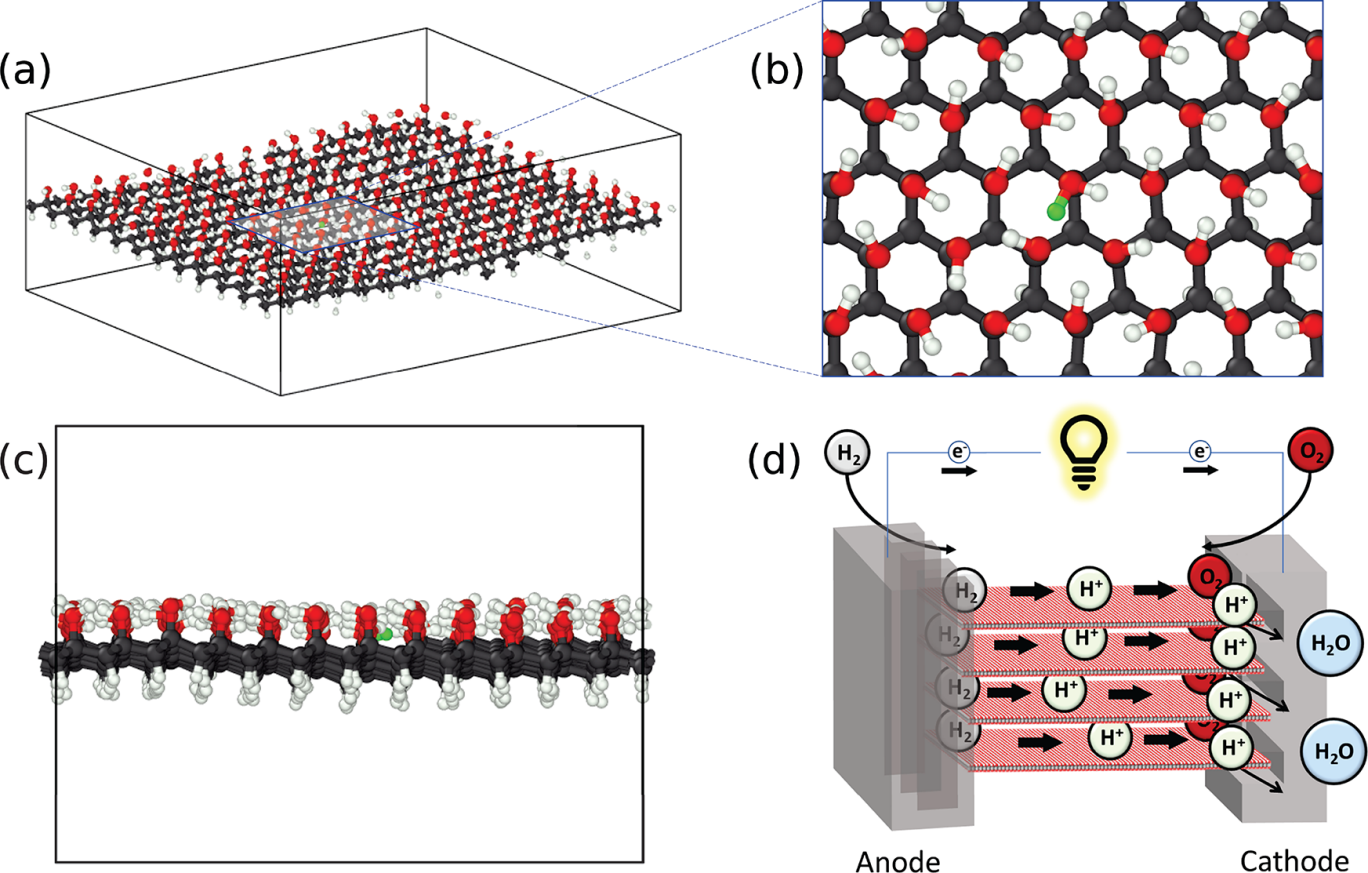
(a) Orthographic view of charged single-sided graphanol containing 961 atoms. C atoms in black, H atoms in white, and O atoms in red. The proton is indicated as a green H atom. (b) Top view of a section containing the proton of panel a, marked by a blue box. (c) Side view of panel a showing hydroxyl functionalization on only one side of the system. (d) Concept diagram of a graphanol based fuel cell stack, wherein H_2_ gas is sent to the anode and O_2_ gas is sent to the cathode. Protons (H^+^) travel from the anode to the cathode to generate electricity and H_2_O is generated as a byproduct at the cathode.

In this work we consider graphanol^43−46^ (Figure 1a–c), an sp^3^ hydrogenated version of graphene (graphane)^47^ functionalized with hydroxyl groups, as a material to facilitate anhydrous PT. Two-dimensional (2-D) sheets of graphanol can potentially be stacked together to form a PEM set up as illustrated in Figure 1d. Graphanol and other functionalized graphane materials, such as graphamine,^48^ were theoretically studied using DFT by Bagusetty et al.^48^ and were found to exhibit anhydrous Grotthuss-like proton conduction. The proton hopping barrier of one-dimensional (1-D) graphanol was estimated to be 60 meV,^43^ which is much lower than Nafion’s barrier of about 360 meV.^49^ As previously noted,^44^ 1-D graphanol is both infeasible to synthesize and undesirable because a single missing OH group defect would effectively block PT. These factors led us to design fully hydroxylated 2-D graphanol. However, estimating diffusion coefficients, barrier heights for PT, or a complete description of the PT mechanism for 2-D graphanol using DFT proved unfeasible. This was due to the inability to simulate atomic trajectories for larger systems and for sufficiently long times because of prohibitive computational costs.

It is important to address the feasibility of synthesizing graphanol, since it is not useful to predict properties of materials that cannot be synthesized in practice. Several forms of hydroxylated graphene have been synthesized over the past several years.^50−56^ The ideal form of graphanol is fully functionalized graphane, with every H atom replaced by an OH group, whereas the materials produced experimentally have varying degrees of hydroxylation. We note that Pumera and co-workers have synthesized a material with a composition of (C_1_O_0.78_H_0.75_)_*n*_,^50^ which is close to the ideal fully functionalized graphanol composition. Importantly, the ideal material is not required to make a practical membrane. PT in graphanol occurs along a triangular lattice. Each node of this lattice is an O atom, and the edges connecting these nodes are possible PT pathways. This triangular lattice has a percolation threshold of 0.5,^57,58^ meaning that graphane that is 50% functionalized with OH groups will conduct protons.

We here study the mechanism for PT in 2-D single-sided graphanol (Figure 1a–c) using atomistic deep learning potentials (DPs) that are trained with DFT data via the deep potential molecular dynamics (DeePMD)^59,60^ formalism. We previously compared the accuracy of the adaptive intermolecular reactive empirical bond order potential (AIREBO) for graphane with our DP and DFT calculations and found that AIREBO lacked the accuracy and simulation stability that DP provides.^61^ We used single-sided graphanol for computational convenience; we envision the real membrane will be double-sided (functionalized on both sides). We use a DP rather than DFT to study PT on graphanol to explore extended time and length scales not accessible with DFT. Our largest graphanol simulations contain over 22 000 atoms and cover nanosecond time scales. We estimate the proton hopping barrier for graphanol to be 99 ± 9 meV, which is significantly lower than for Nafion and most other competing proton conducting materials. We found that the PT mechanism in graphanol involves protons moving along Grotthuss chains, that these chains mutate through OH rotations, and that the overall barrier involves a combination of site-to-site proton hopping barriers and OH rotational barriers. We predict that the effective barrier, and hence the diffusion constant, may be tuned in similar materials by manipulating the hopping and rotational barriers.

## 2 Results and Discussion

### 2.1 Deep Learning Potential Accuracy

It is critical to first establish the accuracy of our DP. We trained the DP using DFT calculations of both uncharged (prefix “u”) and charged (prefix “c”) single-sided graphanol. The charged system contained a single added proton. Hence, a “u24C” graphanol cell is uncharged and contains 24 C atoms, as shown in Figure S1b after DFT structural relaxation. We used the u24C and c24C systems to generate training data to construct DPs, and larger system sizes, such as u/c96C and u/c384C, were used for testing and evaluation purposes. The development of the DP is described in Methods.

**Figure 2:**
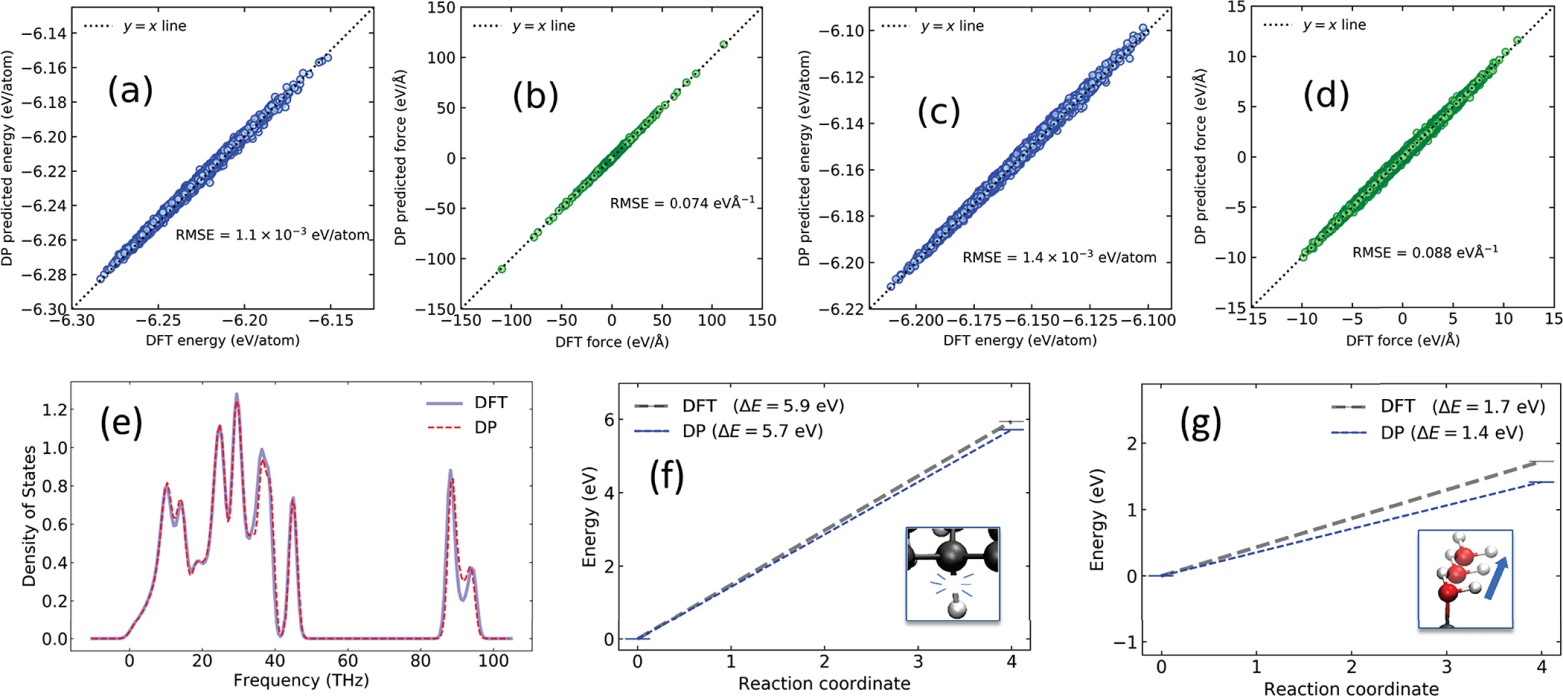
Parity plot of DP-predicted (a) energies and (b) forces for uncharged graphanol compared with values computed from DFT. Parity plot of DP-predicted (c) energies and (d) forces for charged graphanol compared with values computed from DFT. (e) Phonon density of states prediction at 0 K using DP and DFT. (f) C–H bond formation energy from DFT and predicted from DP. (g) H_2_O formation energy from DFT and predicted from DP.

The DP with the lowest energy loss function value from the last iteration of active learning was chosen as the final DP for graphanol. We evaluated our final DP by examining its accuracy in predicting total energies and atomic forces on unseen test data. We used two sets of test data, one for uncharged (u24C) and the other for charged (c24C) graphanol. The uncharged test data was obtained from multiple DFT-MD simulations (at *T* = 1000 K) of u24C structures that were first randomly perturbed and their hydroxyl groups randomly rotated. The charged test data were obtained from two independent DFT-MD simulations of c24C at *T* = 800 K and *T* = 1000 K. Each of these sets of test data contained 4000 snapshots. The root-mean-square errors (RMSE) referenced to DFT values were used as the metric to evaluate the accuracy of the DP; these are shown as parity plots in Figure 2a–d. The RMSE in predicted energies are 1.1 × 10^–3^ eV/atom and 1.4 × 10^–3^ eV/atom for the uncharged and charged sets, respectively. This is within the so-called “chemical accuracy” of 0.04 eV. The corresponding RMSE in predicted forces are 0.074 eV/Å and 0.088 eV/Å, respectively.

Graphanol is expected to have no instabilities in the form of soft modes based on the DFT phonon dispersion spectrum studies of Bagusetty et al.^44^ We computed the phonon density of states (PDOS) of graphanol with our DP for the primitive cell and compared it with the corresponding DFT results in Figure 2e. The phonon calculations were carried out within the quasi-harmonic approximation at 0 K. The agreement between PDOS predicted by our DP compared with DFT calculations over all frequency modes is excellent. DP is seen to slightly overestimate the densities at higher frequencies (around 90 THz). The partial DOS plots (Figure S4) indicate that these modes correspond to the H atoms that are part of the hydroxyl group. We believe that this inaccuracy should not influence our ability to estimate accurate proton diffusion characteristics.

We measured bond formation energies for graphanol using the DP. The proton formation (C–H bond) energy estimated by our DP was 5.7 eV, which is close to the value computed from DFT of 5.9 eV, as shown in Figure 2f. We did not explicitly sample C–H bond breaking events as part of the initial training data to achieve this accuracy. Details regarding generation of training data are provided in Methods and Supporting Information. The H_2_O formation energy (C–O bond energy) for charged graphanol is more crucial for the DP to capture. The DFT bond dissociation energy is 1.7 eV. We explicitly sampled C–O dissociation events in the training data set. The predicted bond energy from DP is 1.4 eV, which is close to that of DFT, as shown in Figure 2g. The initial and final configurations used in computing both formation energies are shown in Figure S6.

### 2.2 Thermal Fluctuations

**Figure 3:**
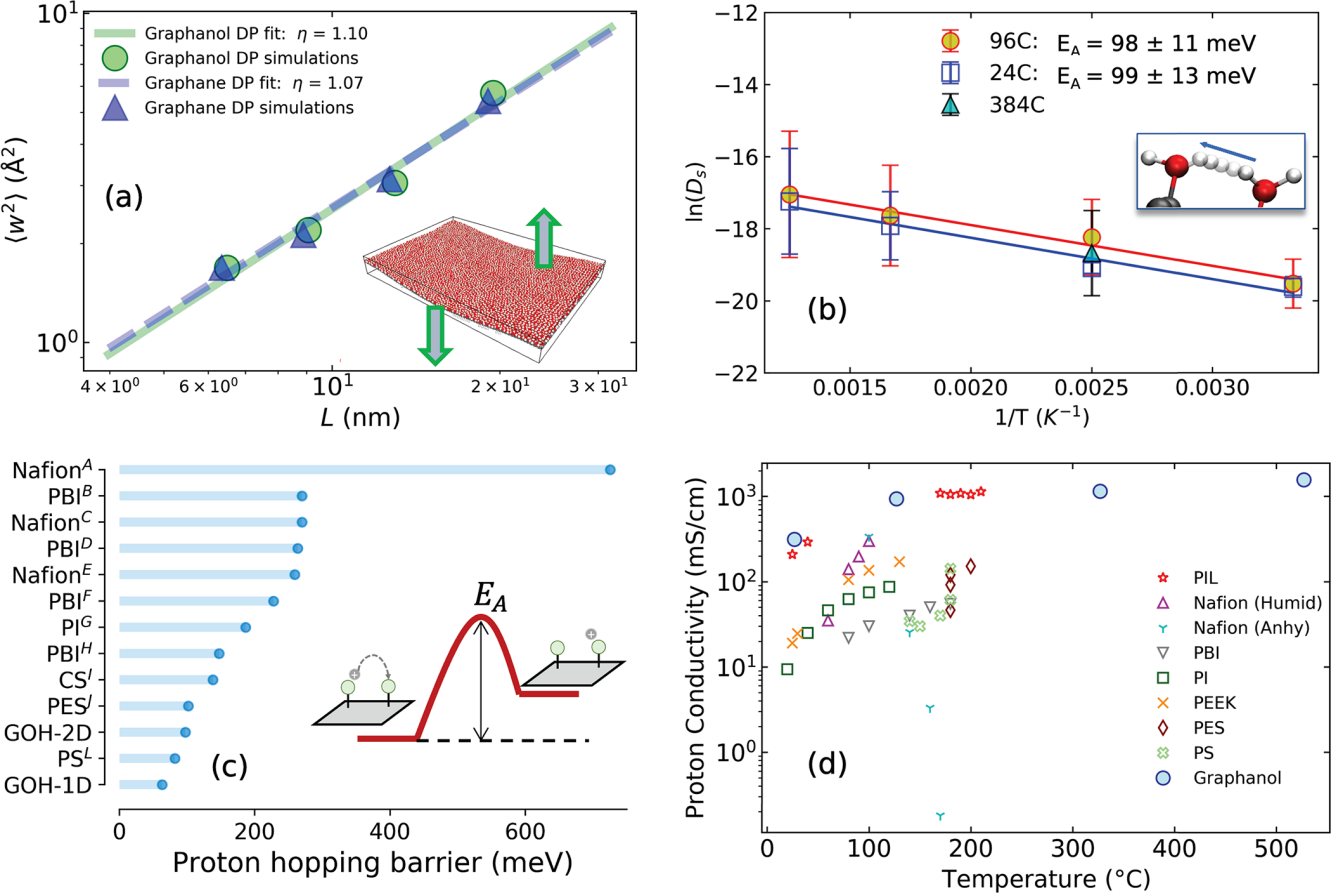
(a) Mean square out-of-plane displacement (a measure of stiffness) of graphanol compared with graphane as a function of system size. (b) Proton diffusion coefficients and Arrhenius equation fits for 24C and 96C system sizes, along with *D*_s_ for 384C at 400 K. (c) The proton hopping activation energies for 1- and 2-D graphanol (GOH) compared with other anhydrous proton conducting materials (sourced from Karimi et al.^25^). Details of the membrane type, meaning of the membrane type superscripts, and the corresponding reinforcement used for other materials are listed in Table S1. (d) Measured proton conductivities for several materials compared with our predictions for 2-D graphanol.

We have calculated the thermal fluctuations in freestanding graphanol as a function of system size to explore its thermal stability, following the approach previously used for graphane and graphene.^61−63^ The squares of the out-of-plane displacements, *w*_*i*_^2^, for each atom *i* were calculated from 50 ps long (0.5 fs time step) MD simulations and used to quantify thermal fluctuations. We measured ⟨*w*_*i*_^2^⟩ from *NVT*-MD simulations run at *T* = 300 K for graphanol at system sizes of 400 to 22 500 atoms and compared with values for graphane, which we calculated for systems having the same number of carbon atoms using our previously developed DP for graphane.^61^ Results are plotted in Figure 3a, where it can be seen that graphanol and graphane have similar ⟨*w*_*i*_^2^⟩ values. The fits to the log transform of the scaling law equation, , are also plotted in Figure 3a. The values of the slopes (a measure of the stiffness), η, are 1.10 for graphanol and 1.07 for graphane, indicating that functionalization of graphane with hydroxyl groups on just one side does not significantly impact its flexibility. This is a surprising result, since we expected that the OH hydrogen bonding network would make graphanol stiffer, resulting in smaller values of ⟨*w*_*i*_^2^⟩. Whether double-sided graphanol will have thermal fluctuations similar to single-sided graphanol remains an open question.

### 2.3 Diffusion Coefficients and Activation Energies

We computed proton self-diffusion coefficients, *D*_s_, on graphanol at four different temperatures (*T* = 300, 400, 600, 800 K) from *NVT*-MD simulations. Fifty independent simulations, each of 125 ps duration, were performed at each temperature to improve the statistics of our calculations. These *D*_s_ values were used to estimate the PT activation energy (*E*_A_) by fitting to the Arrhenius equation. The proton mobility was measured using a center of excess charge (CEC) method, as described below. System size effects were checked by comparing results from c24C, c96C, and c384C system sizes. These results are plotted in Figure 3b, with *D*_s_ values given in Table 1. The *E*_A_ for the c24C system is 99 ± 13 meV, which is statistically indistinguishable from the value of *E*_A_ for the c96C system of 98 ± 11 meV. This indicates that a system size of just 24 carbon atoms is sufficiently large for estimating the PT activation energy (barrier). The average *E*_A_ from the two system sizes is 99 ± 9 meV. As a further test of system size effects, we calculated *D*_s_ for the c384C system at *T* = 400 K. As seen from Table 1, the *D*_s_ values at each temperature for all system sizes agree within the error bars. We also report the *D*_s_ computed from DFT for the c24C system at *T* = 400 K as a test of the accuracy of the DP. The DP and DFT values agree within their combined errors. The value of *D*_s_ can be estimated from a plot of the mean square displacement, MSD, divided by time (see Supporting Information). Plots of the average MSD/(4*t*) vs time are provided in Figure S5. Note that we used a short MD simulation time of 12.5 ps (one-tenth the duration of the DP simulations) for the DFT *D*_s_ calculations because of the computational cost (for comparison, the DP simulations took 45 min of wall time on 1 NVIDIA A100 GPU for 125 ps of simulation time, and the DFT simulation took 245 min of wall time on 96 Intel Xeon Gold 6342 CPU cores (392 CPU hours) for 12.5 ps of simulation time).

**Table 1:** Proton Diffusion Coefficients at Different Temperatures and Different System Sizes (24C, 96C and 384C)^a^

system	*T* (K)	*D*_s_ × 10^8^ (m^2^/s)
24C	300	0.30 ± 0.12
96C	300	0.33 ± 0.21
24C	400	0.59 ± 0.35
24C (DFT)	400	1.16 ± 0.48
96C	400	1.20 ± 0.54
384C	400	0.77 ± 0.62
24C	600	1.65 ± 0.76
96C	600	2.19 ± 0.52
24C	800	3.26 ± 1.53
96C	800	3.95 ± 2.78

a All *D*_s_ values were computed from DP, except the value on the fourth row, which was computed from DFT. The estimated errors are twice the standard deviations.

### 2.4 Comparison with Other Materials

Our calculated value of *E*_A_ = 99 meV is very low compared with typical values for Nafion^49,64^ and not much higher than the barrier estimated for 1-D graphanol of 60 meV.^43^ It is informative to compare the predicted performance of graphanol with other proton conduction materials. We sourced PT activation energies and proton conductivities of several materials from a review paper by Karimi et al.^25^ We plot comparisons of these values in Figure 3c,d. Details for each material are given in Table S1. We note that the results are all experimental except for our predictions for graphanol. We report the activation energies for six classes of materials: Nafion, polybenzimidazole (PBI), chitosan (CS), poly(ether sulfone) (PES), sulfonated poly(ether ether ketone) (SPEEK), and polysulfone (PS). Support-like reinforcements and the use of acidic media are required to facilitate anhydrous proton conduction in some of these materials because they are not intrinsic proton conductors. Graphanol (GOH-2D) has a lower proton hopping activation energy than almost all other materials and is comparable to composite SPEEK with reinforced fluoropolymers and functionalized PS. We used our *D*_s_ estimates at different temperatures to estimate proton conductivities (σ) for graphanol, plotted in Figure 3c. These were calculated via the Nernst–Einstein equation, , where *F* is the Faraday constant, *R* is the gas constant, *T* is the temperature at which *D*_s_ is calculated, and *c* is the concentration of protons in the system. We estimated *c* to be 0.0028 mol/cm^3^ using the c24C system, which corresponds to the number of moles of protons per volume of the membrane (discussed in Supporting Information). We estimated the proton conductivity of graphanol over the temperature range *T* = 300–800 K (27 to 527 °C) to vary between 313 and 1567 mS/cm. Graphanol’s high proton conductivity at low temperature is a major advantage for PEM-FCs because most high temperature PEM-FCs suffer from delayed startup at low temperatures.^65,66^

### 2.5 Proton Transport Mechanism

It is well-known that PT in water, Nafion, and other hydrated materials involves both the Grotthuss mechanism and vehicular transport. In contrast, PT on graphanol must involve only the Grotthuss mechanism, since vehicular transport is not possible. Thus, we expect that a proton on an HOH group (e.g., the green atom in Figure 1b) will hop to one of the six neighboring OH groups, creating a new HOH group. Then, either the initial proton will hop back to the original OH group or the other H atom on the new HOH group will hop, as a “new” proton, to a third OH group. Our hypothesis is that for this process to lead to long-range PT it must also involve OH rotations. This is because the initial set of OH orientations cannot provide pathways to traverse the entire simulation cell. Hence, the apparent value of *E*_A_ must be a convolution of the intrinsic barrier for a proton to hop from one O atom to another O atom and the intrinsic barrier for OH rotation.

**Figure 4:**
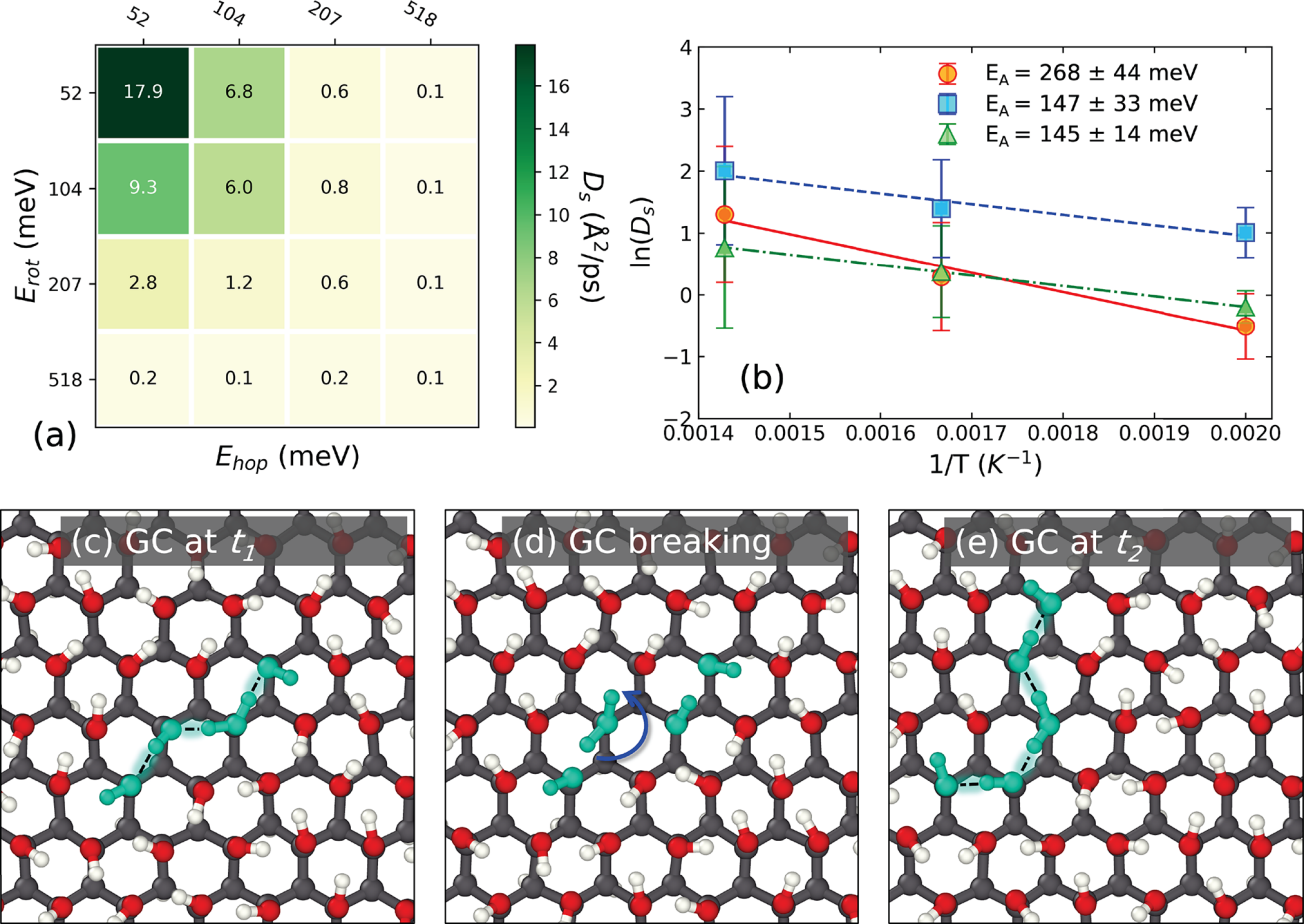
(a) Sensitivity analysis of proton hopping barrier (*E*_hop_) and the hydroxyl rotation energy barrier (*E*_rot_) on proton diffusion coefficient (*D*_s_) using LMC simulations at *T* = 400 K. (b) Sensitivity analysis of *E*_hop_ and *E*_rot_ on apparent PT activation energy (*E*_A_) using LMC simulations at *T* = 500, 600, and 700 K. Arrhenius equation fits of proton diffusion coefficients performed to estimate *E*_A_ at *E*_hop_ = 52, *E*_rot_ = 259 meV (red circles and solid line), *E*_hop_ = 259, *E*_rot_ = 52 meV (blue squares and dashed lines), and *E*_hop_ = 13, *E*_rot_ = 293 meV (green triangles and dash-dot line) (c) Visualization of a Grotthuss chain (GC) formed at some time *t*_1_ in c96C graphanol. Atoms that form the GC are marked in teal and their hydrogen-bonding network is shown as black dashed lines. (d) Rotation of at least one of the hydroxyl groups breaks the previously formed GC. (e) A new GC is formed at some later time *t*_2_.

One way to test our hypothesis that both intrinsic proton hopping and intrinsic OH rotation combine to produce long-range PT is to independently vary the values of the proton hopping barrier, *E*_hop_, and the OH rotation barrier, *E*_rot_, and then observe how the value of *D*_s_ responds to these changes. It is impossible to use DFT or DP simulations for this. Hence, we developed a lattice Monte Carlo (LMC) model of PT on graphanol, which has *E*_hop_ and *E*_rot_ as parameters. Details of the LMC simulations are given in Methods, and details of the algorithm are presented in Supporting Information. We systematically varied *E*_hop_ and *E*_rot_ from 52 to 518 meV and calculated *D*_s_ to perform a sensitivity analysis. These calculations were performed at a constant temperature of *T* = 400 K. The results are tabulated in Figure 4a. We found that both *E*_hop_ and *E*_rot_ impact the value of *D*_s_, with *E*_hop_ having a larger influence on *D*_s_ than *E*_rot_. This can be seen, for example, by noting that for *E*_hop_ = 52 meV and *E*_rot_ = 207 meV we get *D*_s_ = 2.8 Å^2^/ps, whereas for *E*_hop_ = 207 meV and *E*_rot_ = 52 meV we get *D*_s_ = 0.6 Å^2^/ps, which is a factor of 4.7 times smaller. Hence, the impact of hopping and OH rotation is asymmetric. We next computed *E*_A_ for two different barrier combinations: *E*_hop_ = 52, *E*_rot_ = 259 meV and *E*_hop_ = 259, *E*_rot_ = 52 meV, by calculating *D*_s_ as a function of temperature and fitting the data to the Arrhenius equation. These results are plotted in Figure 4b. The overall *E*_A_ appears to be more sensitive to *E*_hop_ than *E*_rot_, as one might expect based on the *D*_s_ sensitivity analysis. The system for *E*_hop_ > *E*_rot_ gave *E*_A_ = 268 ± 44 meV, which is the same as *E*_hop_ = 259 meV within the error of the calculation. The reverse case, *E*_hop_ < *E*_rot_, resulted in a much lower *E*_A_ of 147 ± 33 meV, thus showing that having a low *E*_hop_ is critical to PT. We also carried out LMC calculations for the values of hopping and rotational barriers estimated for graphanol, *E*_rot_ = 293 and *E*_hop_ = 13 meV, from which we estimated *E*_A_ = 145 ± 14 meV. This value is larger than the *E*_A_ estimated for graphanol from our DP simulations, but given the approximate nature of the LMC model, the agreement is reasonable. This indicates that our LMC approach is able to capture the essential physics of PT on graphanol.

Note that for graphanol *E*_rot_ ≫ *E*_hop_, which means that hopping will occur rapidly compared with rotation; this is indeed what we observe from visualization of a c96C MD simulation at 600 K, which is included in Supporting Information. If the magnitudes of the barriers were reversed, one would expect that rotations would be rapid and that proton hopping would be rare events. Given the slow rotation relative to hopping, we propose the following expectations for the detailed PT mechanism: (1) the low value of *E*_hop_ means that protons will readily hop back and forth between a pair of O atoms, which we call “rattling”; (2) at any given time a proton may be part of a Grotthuss chain (GC), which we define as a chain of 3 or more OH groups connected by hydrogen bonds arranged such that a proton can move forward and backward along the chain without any OH rotations; (3) the length of a GC is finite and unlikely to include more than about 10 OH groups; (4) GCs can break and form new GCs through OH rotations; (5) the GCs are long-lived compared with the time required for proton to hop from one O to a neighboring O; multiple hops along the chain will therefore typically take place before the chain breaks and a new chain is formed.

Qualitatively, our GC hypothesis predicts that a proton spends considerable time rattling between pairs of O atoms and traversing part of the GC to which it belongs. These events do not lead to long-range PT. However, new GCs are formed when one or more OH groups that are part of the GC rotate to make new hydrogen bonds. It is this process of breaking and forming GCs that leads to long-range PT.

**Figure 5:**
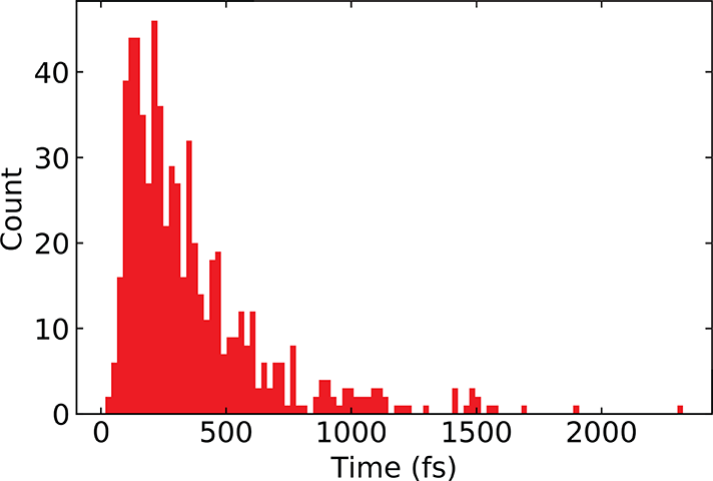
Traceback time histogram calculated by measuring the amount of time a proton takes to come back to its starting O atom. Data contains multiple pathways that include different number of distinct O atoms involved in each path.

Snapshots of a GC breaking and forming event observed in our simulations are shown in Figure 4c–e. We now address the question of how to test our GC hypothesis. It is a challenging task to unambiguously and automatically identify GCs. We adopt the following approach. If a proton begins its diffusion path at a given O atom, traverses at least 2 other O atoms and then returns to the starting O atom we label this a “traceback” event. A traceback of length *N* (i.e., containing *N* O atoms) is sufficient (but not necessary) proof of the existence of a GC of length *N*. We have measured the traceback time, which is the time required for a CEC to traverse the length of the GC and return to the original O atom. We use the traceback time as a surrogate for the GC lifetime in our analysis, recognizing that a less restrictive definition of a GC (not requiring a traceback event) may have significantly shorter lifetimes. A plot of the histogram of traceback times for *N* = 3, 4, 5, 6 is given in Figure 5. The traceback times follow a log-normal distribution. The log transform of the data (which is Gaussian) is shown in Figure S9, where we also show the histograms for individual GC lengths. The mean traceback time was calculated to be 240 fs. This does not mean that the average lifetime of a GC is close to this value. It means that some of the GCs have lifetimes long enough to enable traceback events, and these events take, on average, about 240 fs. Nevertheless, it does prove the existence of GCs and that they can have lifetimes that are relatively long compared to proton hopping events over multiple OH groups.

An animation showing the movement of the CEC that qualitatively demonstrates the dynamic nature of the GCs is provided in Supporting Information. The animation shows the positions of the oxygen atoms associated with the CEC as a function of time, with a history showing the positions of the CEC over the previous 125 fs. The simulation is for the c96C system at 800 K and shows 9 ps from a 125 ps simulation. The trajectory traced out over the entire simulation is shown in light blue. The CEC positions over the past 125 fs are shown in dark blue.

## 3 Conclusion

We have computed proton diffusion coefficients, diffusion activation energies, and estimated proton conductivities for graphanol, a proposed material for anhydrous proton conducting membranes. We have shown that anhydrous proton diffusion on graphanol is facile with an activation energy of 99 meV, which is lower than most other proton conducting materials. We predict that graphanol is a very promising material for PEM fuel cells and should be the subject of further experimental and theoretical work.

We have identified the details of the proton transport mechanism in graphanol. The mechanism involves a combination of proton hops from one OH group to neighboring OH groups and OH rotations. We show that the protons hop along Grotthuss chains, which consist of a pathway of hydrogen bonds allowing proton conduction forward and backward along the chain without any OH rotations. Long-range proton transport is accomplished through formation of new Grotthuss chains that are created when OH groups rotate and form new networks of hydrogen bonds. Hence, the apparent diffusion activation energy is a convolution of the intrinsic proton hopping barrier and the intrinsic OH rotation barrier. We carried out sensitivity analysis using a lattice Monte Carlo model, and we found that the effective barrier is more sensitive to the hopping barrier than the rotation barrier.

Our findings concerning the details of the proton diffusion mechanism on graphanol indicate possible approaches to decreasing proton diffusion activation energies. For instance, missing OH groups can be introduced to lower the local OH rotational barrier without impacting the hopping barrier. In fact, actual materials are likely to exhibit missing OH groups and, given that the percolation threshold is 0.5, proton conductivity can still take place even when nearly half of the OH groups are replaced by H atoms. Another possibility is to pattern the surface with alternating bands of OH and H functionalizations, which is likely to exhibit lower barriers for OH rotation at the band edges. We note that Walton et al.^67^ have demonstrated the ability to control spatial functionalization of graphene, indicating the feasibility of producing banded graphanol.

The proton transport mechanism we have identified for graphanol can be extended to other 2-D hydrogen bonded networks, such as graphamine, which has been previously, but incompletely, studied using DFT.^48^ The detailed model of proton transport mechanism developed in this work allows us to propose potential ways to optimize the proton diffusion activation energy, not only for graphanol, but also, potentially, for similar classes of materials. For example, a substrate other than graphane could be used to produce larger OH–OH distances, which would decrease the rotational barrier. However, this can also increase the hopping barrier. A careful optimization of the OH–OH distance may therefore be required to achieve sufficiently low rotational barriers. The functional groups could also be varied, to promote the formation of 2-D Grotthuss networks, rather 1-D than chains. This can provide a means to support long-range proton hopping with no need for rotations of the functional groups. Future work will explore proton conduction on graphanol that has been functionalized on both sides, the role of defects, and the impact of stacking layers of graphanol.

## 4 Methods

### 4.1 DFT Methods

DFT calculations were performed to optimize the cells of graphanol, generate training data for the DPs, and produce reference data to validate the DPs. The Vienna ab initio simulation package (VASP)^68−71^ was used to carry out the DFT calculations. Electron–ion interactions were described using the projected augmented-wave (PAW) method.^72^ An energy cutoff for the plane-wave expansion of 520 eV was used, and the total energy convergence for self-consistent field calculations was set to 10^–6^ eV. A k-point mesh not containing the Γ-point with a spacing of 0.4 Å^–1^ was used. We used the generalized gradient approximation exchange-correlation functional of Perdew–Burke–Ernzerhof (PBE).^73,74^ Further details are included in Supporting Information.

### 4.2 Center of Excess Charge

We developed a modified center of excess charge (CEC) method, based on previous work by Li and Swanson,^75^ to estimate the diffusion of protons on charged graphanol. Similar methods, such as the proton indicator,^76,77^ have also been developed to model excess protons in bulk water using only geometric information. Our CEC method is a classical surrogate for the position of the proton that tracks the position of O atoms with two H nearest neighbors, thus estimating the location of charge centers (*r*_CEC_). PT self-diffusivities were computed from the diffusion of the CEC. More details regarding the CEC method are given in Supporting Information.

### 4.3 DP Training

**Figure 6:**
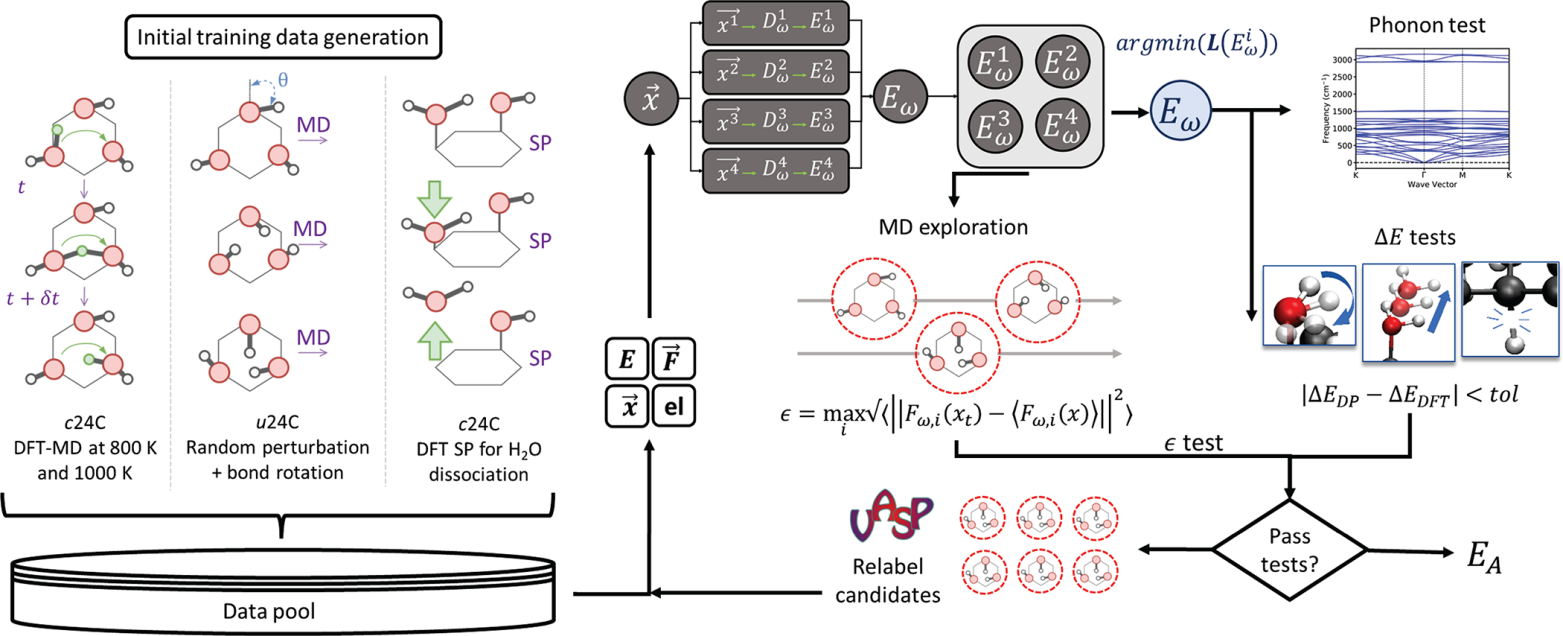
Active Learning-based training schematic. First, initial training data were generated using charged and uncharged graphanol structures. Three sets of initial data were generated based on specific criteria (left): proton hopping, hydroxyl group rotation, and H_2_O dissociation. An ensemble of DPs (indicated by *E*_ω_^1^, *E*_ω_^2^, *E*_ω_^3^, and *E*_ω_^4^) was developed to enable DP testing and data relabeling. The best DP was used to perform phonon tests, bond energy tests, and MD exploration. The other DPs in the ensemble were used to identify relabeling candidates (right). The DP was accepted if it passes all tests; if not, identified configurations from the MD exploration were relabeled with DFT and added back to the training set.

The process of training and evaluating DPs is illustrated in Figure 6. Each DP was fitted to training sets consisting of DFT calculations on the periodic cells of graphanol. Each DP contains an embedding and a fitting neural network (NN) that uses atomic coordinates as input and gives total energies and forces as output. The total energy of the system was computed as the sum of individual atomic energies. The energy of an atom *i* was calculated based on the number of neighboring atoms within a cutoff radius. We set a cutoff radius of 6 Å with a smoothing cutoff of 2 Å for the atomic coordinates, which were used to construct descriptors. The fitting NN used these descriptors as input. The training data set was distributed among several batches to train each DP.

We employed an active learning approach to develop improved DPs for graphanol with the help of the deep generator (DP-GEN) scheme of Zhang et al.^78^ The training contained a series of iterations that was designed to improve the quality of the DP by exploring a larger PES. An ensemble of DPs was trained from the same data set for each iteration. The only difference in the DPs was the values of the initial NN model parameters (ω). Each iteration generated sufficiently diverse data to minimize the variance between the ensemble DPs after training. Thus, the variance in forces was used as an error indicator (ϵ) to classify DP predictions to be selected for relabeling. A schematic of our DP generation methods is provided as Figure 6. A detailed description of the data generation, DP training, and validation procedures is provided in Supporting Information.

### 4.4 Lattice Monte Carlo

A LMC model was constructed to help us address questions regarding the PT mechanism. We generated an undirected graph to simulate the c24C system using the Networkx package.^79^ Each node in the graph is a potential H atom site, and each edge defines where any H atom at a node can move. Each node has three edges, thus making a hexagonal lattice structure. Selected hexagons are counted as O atom centers if its neighboring O atom is one edge length away. The graph for c24C is depicted in Figure S10. H atoms can move around a hexagon to simulate OH rotation. H atoms can also move to an adjacent hexagon that is connected by an edge to simulate a proton hop. Moves are decided based on the following constraints: 1. Rotations are attempted only if the number of the randomly picked O has one H attached, else a proton hop is attempted. 2. Rotation/hopping attempts are rejected if (a) the selected H atom has no empty adjacent sites, (b) if the site to which the H atom will move has occupied neighbors. 3. If 2(a) and 2(b) are not true, then a random number ζ is picked from a uniform distribution *U*(0, 1). Rotation/hopping is accepted if ζ is less than their corresponding Boltzmann factors, else it is rejected. Any oxygen atom (hexagon) that has two H neighbors (filled nodes) is considered the CEC, and its geometric location is stored, which is then used to estimate the PT diffusion coefficient. Further details on using these graph models to run LMC simulations are discussed in Supporting Information.

## Data Availability

DP for graphanol, python code to compute CEC, python code to perform lattice Monte Carlo simulations, and initial configuration of graphanol. https://github.com/JKarlJohnson/protonConduction_graphanol

## Supporting Information Available

Visualization of graphanol cells, details of DFT, details of CEC, plots of projected phonon density of states, MSD/(4*t*) plots, visualization of configurations used to compute bond formation energies, details regarding proton hopping energy and hydroxyl group rotation energy, details about proton concentration calculation, comparison of proton conducting materials, details about lattice Monte Carlo simulations, and results of CEC traceback events in graphanol (PDF)
Animation showing a 1.25 ps clip from a 125 ps DP-MD simulation of c96C at 600 K (MP4)
Animation showing the dynamics of Grotthuss chains via tracing the history of the CEC (MP4)
