# Supplementary material for: In Silico Demonstration of Fast Anhydrous Proton Conduction on Graphanol
Source: ACS Appl Mater Interfaces. 2023 May 16;15(21):25873–83. doi: 10.1021/acsami.3c04022 (PMC10236431; doi:10.1021/acsami.3c04022)
Supplement: Supplementary file 1 — am3c04022_si_001.pdf [file am3c04022_si_001.pdf]

Supporting Information: In Silico Demonstration of Fast Anhydrous Proton Conduction on Graphanol

Siddarth K. Achar,[†,‡] Leonardo Bernasconi,[¶] Ruby I. DeMaio,[‡] Katlyn R. Howard,[‡] and J. Karl Johnson[*,‡]

†*Computational Modeling & Simulation Program, University of Pittsburgh, Pittsburgh, Pennsylvania 15260, United States*

‡*Department of Chemical & Petroleum Engineering, University of Pittsburgh, Pittsburgh, Pennsylvania 15261, United States*

¶*Center for Research Computing and Department of Chemistry, University of Pittsburgh, Pittsburgh, Pennsylvania 15260, United States*

E-mail: karlj@pitt.edu

Graphanol Cells

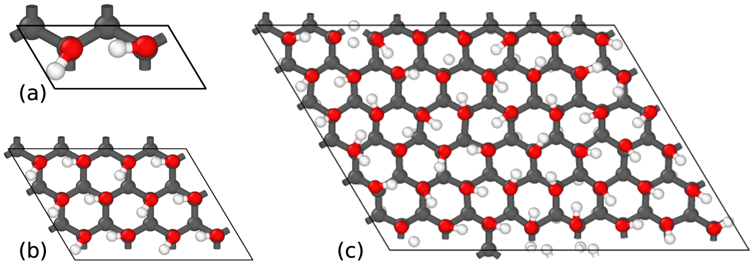

Figure S1: Top views of (a) unit cell, (b) u24C cell, and (c) u96C cell of single-sided graphanol.

DFT Method Details

Density functional theory (DFT) data were generated using the Vienna *ab initio* simulation package (VASP). We used the projected augmented-wave (PAW) method[1] to describe electron-ion interactions and set a plane-wave cutoff of 520 eV. The generalized gradient approximation (GGA) exchange-correlation functional of Perdew-Burke-Ernzerhof (PBE)[2,3] was used. The Brillouin zone sampling was performed using a Monkhorst-Pack k-point grid size of $3 \times 3 \times 1$ for the 24C cells. We determined this to be the optimal grid size as shown below. The ionic positions were relaxed until the forces were lower than the tolerance of 10^{-3} eV/Å for optimization calculations. We used a vacuum spacing of 20 Å above the surface of graphanol to mitigate interactions between layers of the material under periodic boundary conditions. A u24C cell was transformed into a c24C cell by the introduction of an additional proton to the hydroxylated surface, giving a system with a $+1e$ charge. Calculations on charged systems under periodic boundary conditions are commonly handled with a homogeneous neutralizing background jellium charge. This background charge was shown to have a very small influence on proton dynamics.[4]

Training data were generated using these DFT settings for the u24C, c24C and u96C systems. We employed both DFT-MD and single-point VASP calculations generate a suf-

ficiently varied training dataset. DFT-MD simulations in the NVT ensemble were carried out using a Nosé-Hoover thermostat.[5] The frequency of temperature oscillations were set to 40 time steps (SMASS=0).

k-point Mesh Optimization

We determined the optimal number of k-points required to converge the total energy of graphanol by running multiple single-point VASP calculations. We considered two sets of k-points, only one of which included the Γ point. Each set comprised ten different calculations in which the number of k-points was progressively increased by varying the VASP KSPACING tag from 0.1 to 1.0 in increments of 0.1. KSPACING is defined as the smallest allowed spacing between k points and takes the units of Å^{-1}. The results are shown in Figure S2. We chose a KSPACING of 0.4 with the gamma point not included to be the best option. This is not necessarily the optimal case, because the energy does not vary monotonically with the number of k-points. However, a spacing 0.4 seems to be accurate enough compared to the fully k-point converged calculation. This spacing corresponds to a Monkhorst-Pack k-point grid size of $3 \times 3 \times 1$.

Center of Excess Charge (CEC)

Proton transport (PT) was evaluated by tracking the center of excess charge (CEC) in charged graphanol. The CEC of a configuration is the position of the oxygen atom that contains a proton, thus carrying an excess charge. The CEC is defined as the oxygen atom that has two H nearest neighbors (cutoff radius of 1.2 Å). It is possible, because of fluctuations in the O−H bond distances, to encounter situations where there are multiple O atoms having two nearest H neighbors, even when there is only 1 proton in the system. When this arises and when we have no history of where the CEC was at the previous time step (such as when starting the CEC calculation after equilibration), then we assign the CEC by comparing the

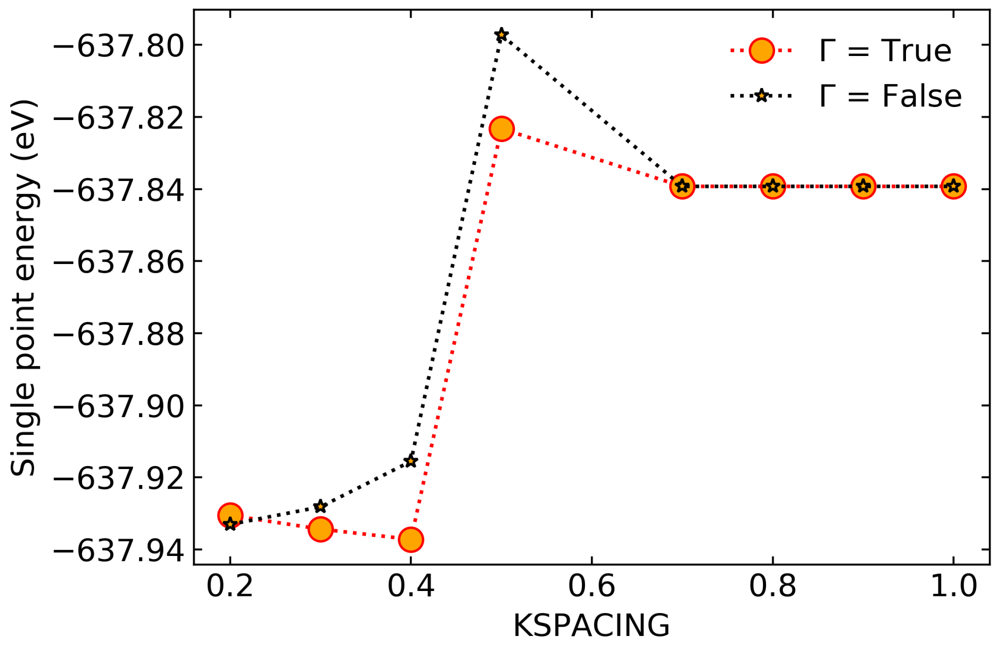

Figure S2: Convergence of energy with the spacing between k-points. KSPACING was varied from 0.2 to 1.0 with (orange circles) and without (black stars) Γ point.

sum of the O−H distances. The O atom with the lowest sum of O−H distances is assigned as the CEC. This is illustrated in Figure S3a, where the O atom in the green circle is the CEC for the given configuration. The sum of the O-H and O-H^+ bond distances is indicated as $\Sigma d_{\mathrm{O-H/H^+}}$. A constraint was applied that requires the CEC at time $t + \delta t$ during an MD simulation to be within the first nearest O neighbors of the CEC at time t. This is shown in Figure S3b where the potential CECs for $t + \delta t$ can be A, B, C, D, E, F or G. In other words, in one time step the CEC must either remain on the original O atom or hop to one of its 6 nearest neighbors. This constraint is realistic because protons cannot hop farther than a nearest neighbor O atom within a single MD time step. PT self-diffusivities (D_s) were computed from multiple independent production runs using Einstein's relation,

$$D_S = \frac{1}{2td}\langle \sum |r_{CEC}(t) - r_{CEC}(0)|^2 \rangle \tag{S1}$$

where t is the time, $d = 2$ is the dimensionality of the system since the diffusion is 2-D, r_{CEC} is the position of the O atom that is labeled as the CEC at time t. It is important to note that these graphanol systems have only a single added charge and thus will only have a single CEC position at any given time. Running multiple production simulations was required to achieve improved statistics in our results. The uncertainties in D_s are reported as twice the standard deviation of the independent values. The calculated D_s at different temperatures were used to measure the PT diffusion energy barrier (E_A) using the Arrhenius equation,

$$\ln(D_S) = \ln(D_0) - \frac{E_A}{RT}. \tag{S2}$$

Other CEC definitions have appeared in the literature. For instance, Li and Swanson[6] have proposed a rigorous procedure for tracking the evolution of the CEC position in DFT-MD simulations, which accounts for the potential coexistence of multiple proton migration paths in complex environments.[7] The CEC tracking method we propose here provides a simple alternative, for the case of graphanol, to the approach of Li and Swanson.[6] Our method relies exclusively on geometric information concerning the arrangement of the O and H atoms and their neighbor atoms during a dynamical simulation and does not require an analysis of potential competing bonding topologies.

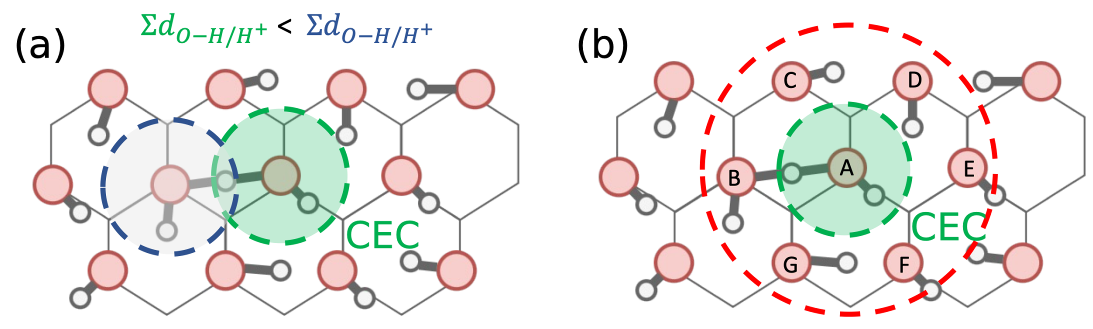

Figure S3: (a) Schematic representation of the approach used to locate the CEC for a given static graphanol system. (b) Schematic demonstrating that the CEC for time $t + \delta t$ can only lie within the dashed red circle, i.e., nearest O neighbors.

Projected Phonon Density of States

Projected phonon density of states for the primitive cell of 2-D single-sided graphanol (u2C) were computed with DP and compared with VASP, as shown in Figure S4.

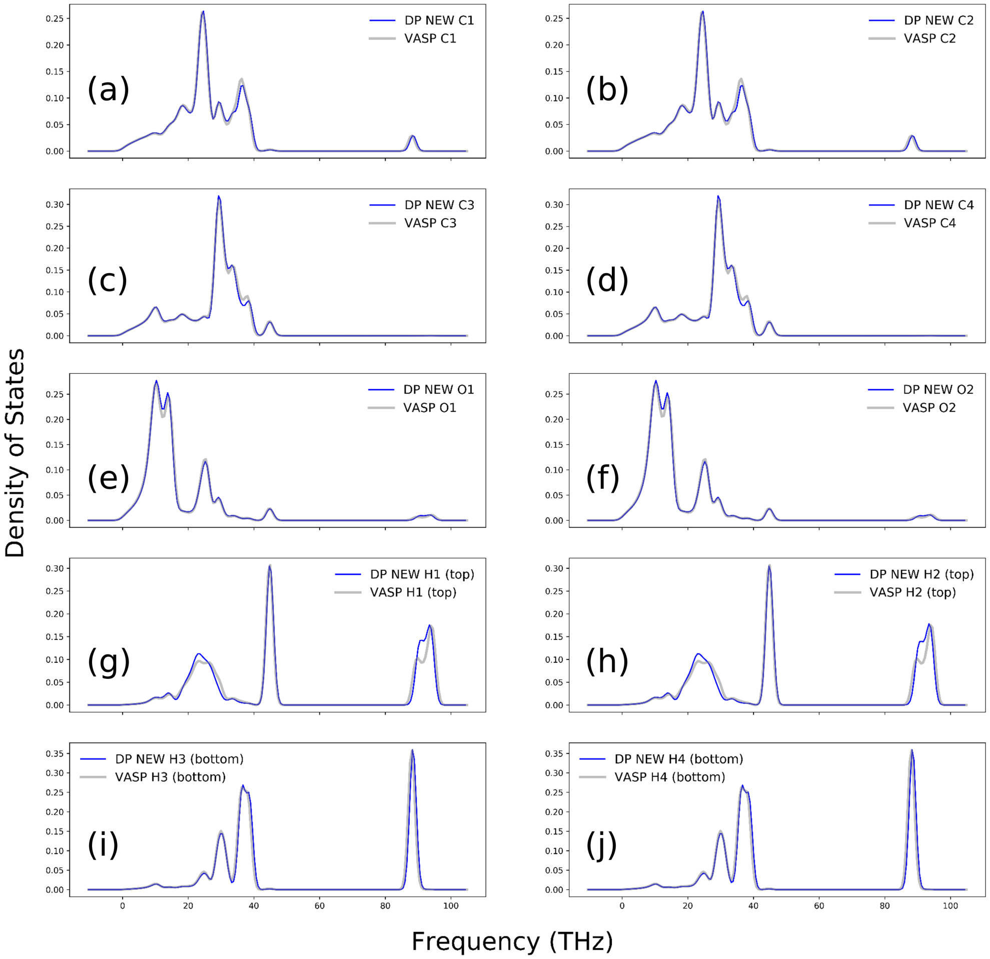

Figure S4: Projected phonon density of states for uncharged graphanol (u2C). (a-d) Modes from C atoms, (e-f) modes from O atoms, (g-h) modes from H atoms on top the u2C surface attached to the hydroxyl group, and (i-j) modes from H atom on the bottom of u2C attached to C atoms.

Mean Square Displacement Plots

We have computed the mean square displacements of the CEC, which is the ensemble averaged term in Eq. S1, $MSD(t) = \langle \sum |r_{CEC}(t) - r_{CEC}(0)|^2 \rangle$. We used multiple time origins

in these calculations. We see from Eq. S1 that a plot of $MSD(t)/t$ must approximately be a straight line with zero slope at long times if the system obeys the Einstein diffusivity relation. If $MSD(t)/t$ consistently decreases with time then the system is sub-diffusive (as is seen for systems that obey single-file diffusion) and if it increases with time the system is super-diffusive (e.g., ballistic diffusion). Plots of the $MSD(t)/(4t)$ are provided in Figure S5. The factor of 4 is $2d$, where $d = 2$ is the dimesionality of the problem, so that $MSD(t)/(4t)$ gives an estimate of D_s if it is approximately straight line with zero slope. We see from Figure S5 that all the plots are very nearly flat, after the initial part, as one expects for Fickian diffusion. These plots prove that our simulations are sufficiently long to give well-developed diffusive behavior.

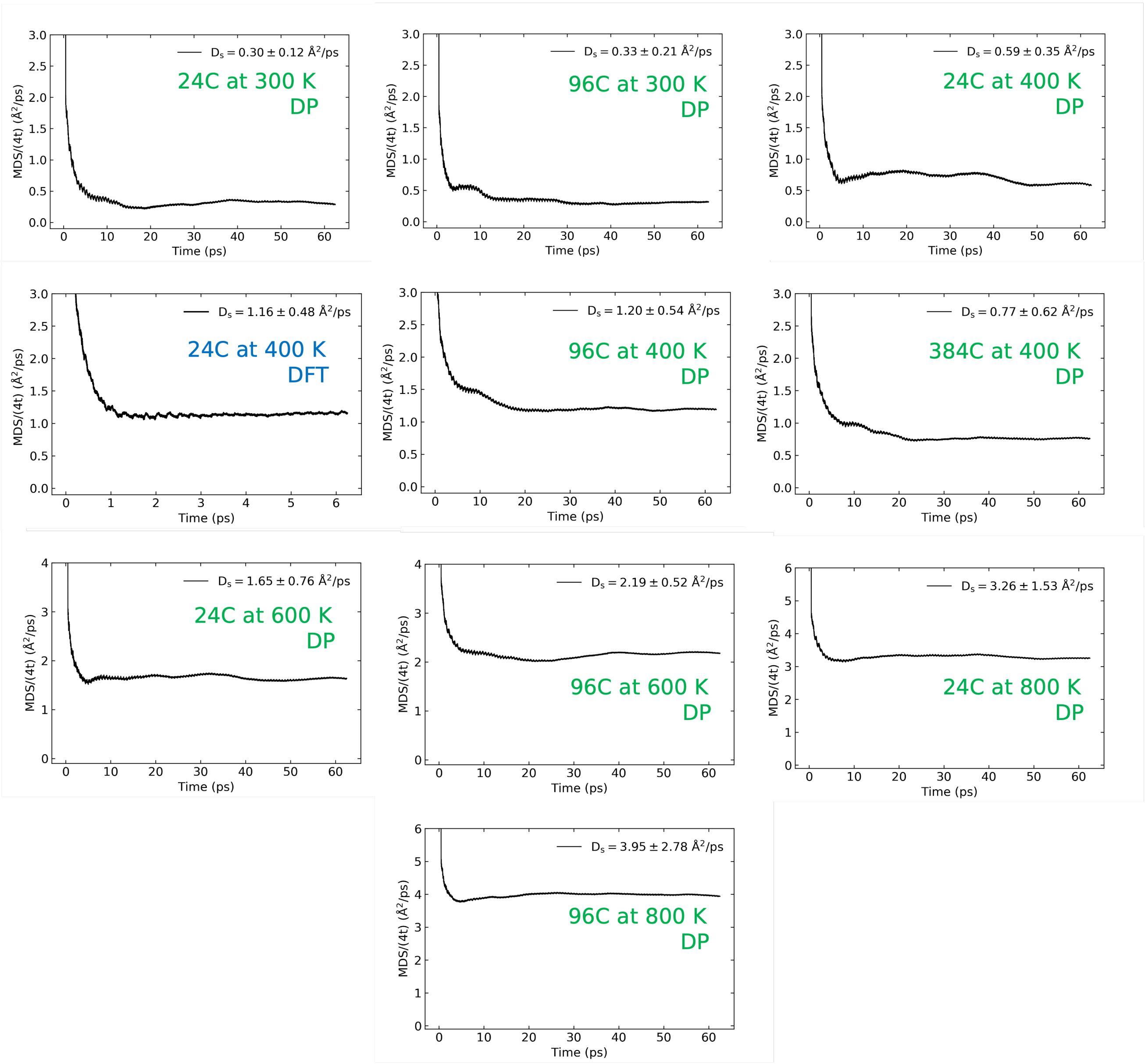

Figure S5: MSD/(4t) plots with average diffusion coefficient values (D_s) for different system sizes and at different temperatures, as listed in Table 1.

Bond Formation Energy Configurations

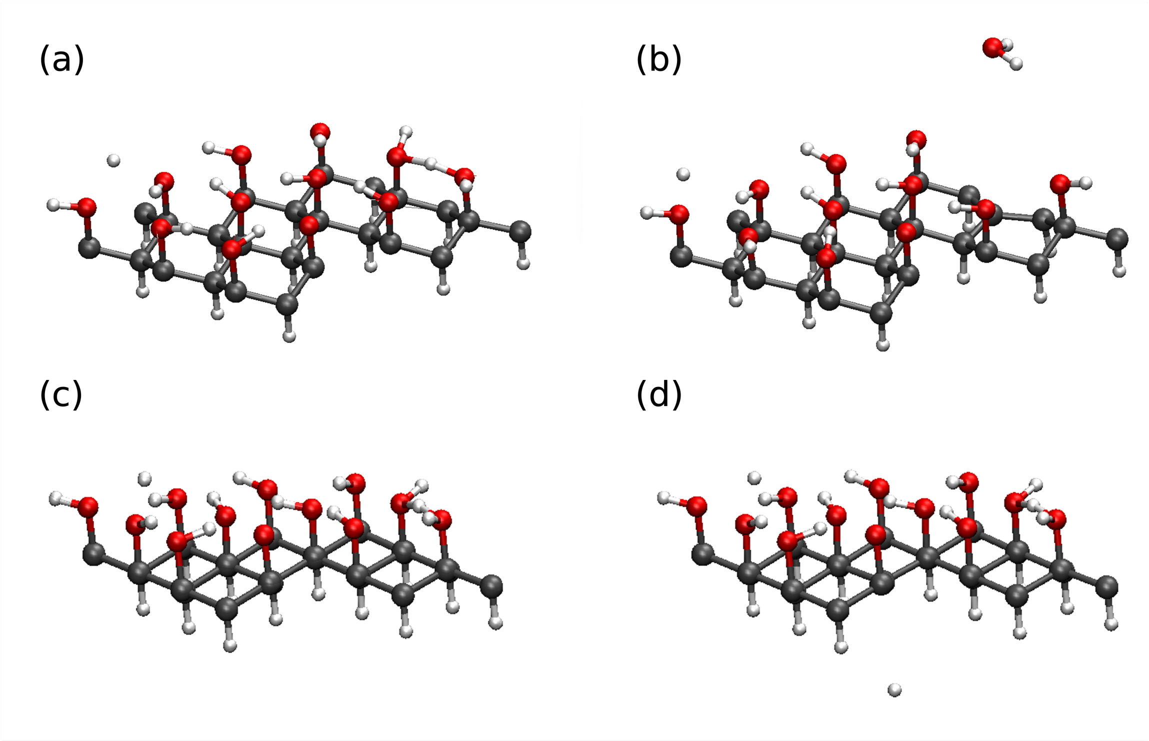

Figure S6: (a) Initial and (b) final configurations used in the calculation of the H_2O formation energy. (c) Initial and (d) final configurations used in the calculation of the proton formation energy (C−H bond dissociation energy).

Proton Hopping Energy

The proton hopping energy, E_{hop}, is defined as the intrinsic activation energy for a single proton to hop from one O atom to a neighboring O atom. E_{hop} is expected to be significantly smaller than the overall PT barrier E_A. We used our final DP to estimate the value of E_{hop}. Two images from a c24C DP-MD simulation were taken and geometrically relaxed using the DP. These two images constitute the start and end state of a proton hopping event, as shown in Figure S7. We then performed a geometric interpolation to identify 11 intermediate states between the start and end images. Single-point energies at $T = 0$ K were calculated for start, end and these 11 intermediate images. The energy required for a proton to move

from the start state to the maximum energy transition state is 12.25 meV. The barrier for the reverse transition is 13.64 meV. The average of the two barriers is 12.94 meV, which we take as an estimate of E_{hop}. This energy is about one order of magnitude smaller than the overall PT barrier, E_A. We note that our calculated value of E_{hop} is similar to the reported barrier for proton hopping in water, computed from potential of mean force.[8]

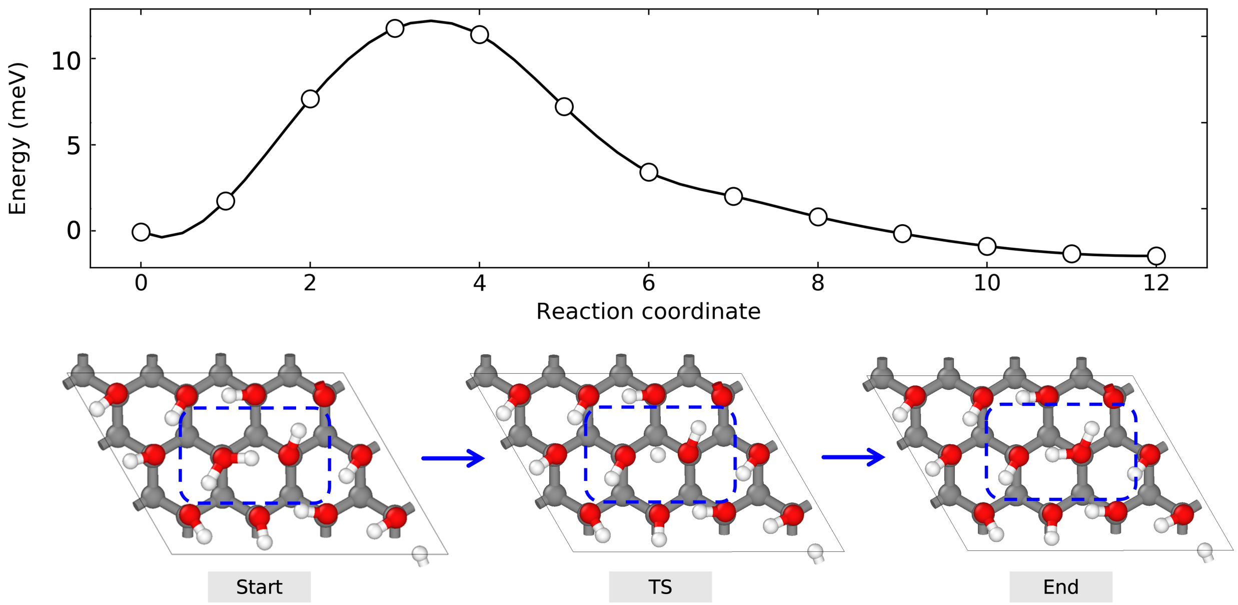

Figure S7: Estimate of the energy barrier for a single proton hopping event (E_{hop}) using the DP. All energies are estimated by performing single point calculations at $T = 0$ K. Visualization of the proton hopping event in shown at the bottom. The location of the proton changes from the "Start" state to the "End" state. A blue dashed box is added to mark the position where the proton hopping is occurring.

Hydroxyl Group Rotation Energy

Calculating the activation energy for hydroxyl group rotation in graphanol, E_{rot}, is challenging because the rotation of each hydroxyl group is coupled to the rotation of other groups. We therefore calculated *rotational diffusion* coefficients, D_r,[9] as a function of temperature as a way to estimate E_{rot}. Angular mean-squared displacements (AMSD) $\langle \phi^2 \rangle$ were first computed by tracking the rotation of dihedrals of all the OH groups from DP-MD simulations.

The C-C-O-H chain forms the dihedral ϕ_i for the OH group i, as shown in Figure S8a. The rotational diffusion coefficient D_r was calculated using the Einstein relation,

$$D_r = \frac{(\overline{\Delta\phi})^2}{2t} \tag{S3}$$

where $(\overline{\Delta\phi})^2$ is the time averaged AMSD and t is the simulation time. We used the DP to run several 2.5 ns NVT-MD simulations at three different temperatures T = 800, 900 and 1000 K. We ran 20 independent runs for each temperature to obtain improved statistics. We then estimated E_{rot} by fitting the D_r to the Arrhenius equation,

$$\ln(D_r) = \ln(D_0) - \frac{E_{\text{rot}}}{RT}. \tag{S4}$$

The fitting gives $E_{\text{rot}} = 293\pm\ 45$ meV, which is higher than our estimates for E_A and significantly higher than our estimate for E_{hop}.

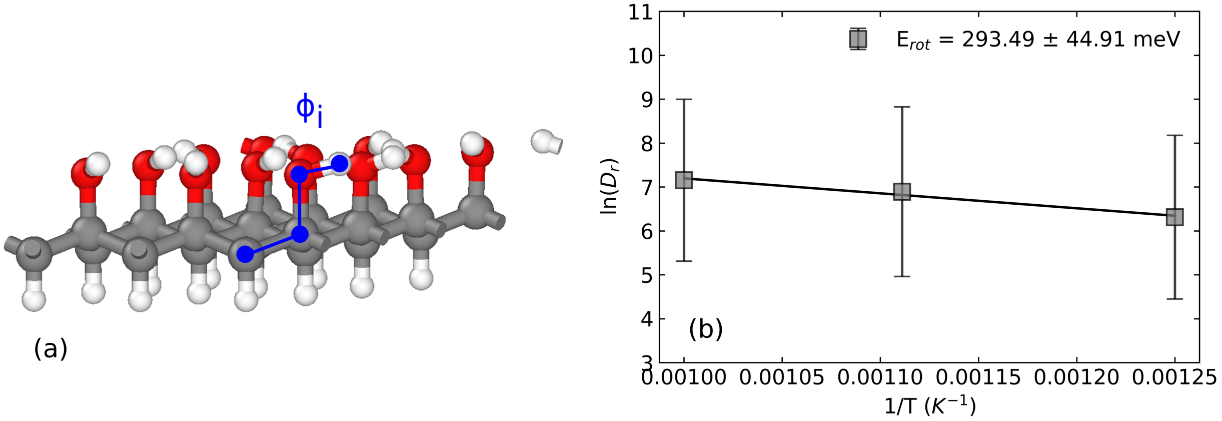

Figure S8: (a) Dihedral angle (ϕ_i) formed by a hydroxyl group i and two C atoms. The C-C-O-H connection is shown in blue. (b) The Arrhenius plot for D_r and the corresponding fit to obtain E_{rot}.

Proton Concentration in Graphanol

The concentration of proton (c) was used to calculate proton conductivities (σ) of graphanol, from the Nersnt-Einstein equation. We estimated c, the number of moles of protons per volume, based on the c24C system. A single added proton corresponds to 1.67×10^{-24} moles of protons. The height of the graphanol cell after optimizing the c axis is about 6 Å. We accounted for non-optimal layer packing by increasing the value of c axis by 2 Å. The cell lattice vectors (in Å) are (10.3, 0.0, 0.0), (-3.9, 6.8, 0.0), (0.0, 0.0, 8.0), which givs a volume of 6×10^{-22} cm^3. This results in a c value of 0.0028 mol/cm^3.

Comparison of Proton Conducting Materials

Table S1: Membrane types, labels used in Figure 3c, reinforcements material used, and the activation energy for PT in meV.

Membrane type	Figure 3c Labels	Reinforcements	Activation Energy (meV)
Graphanol (2D)	GOH-2D	-	99
Graphanol (1D)	GOH-1D	-	63
Nafion (Composite)[10]	$Nafion^{E}$	-	259
Nafion (Nanocomposite)[11]	$Nafion^{C}$	POSS	269
Nafion (Blend)[12]	$Nafion^{A}$	Poly (1-vinyl-1, 2, 4-triazole)	725
Polybenzimidazole (Copolymer)[13]	PBI^{B}	3,5-pyridine-r-2OH-PBI, 3,5-pyridine-r-para-PBI, and 3,5-pyridine-r-meta-PBI	270
Polybenzimidazole (Sulfonated)[14]	PBI^{D}	SO_3H groups	263
Polybenzimidazole (composite)[15]	PBI^{B}	Acidic surfactant	147
Polybenzimidazole (Blend)[16]	PBI^{F}	Lignosulfonate	227
Polyimide (Acid doped)[17]	PI^{G}	H_3PO_4	186
Polyether sulfone (composite)[18]	PES^{J}	Amino-functionalized mesoporous silica	102
Polysufone[19]	PS^{K}	2,4,6-tri (dimethyl aminomethyl)-phenolt	82
Chitosan (Nanocomposite)[20]	CS^{I}	phosphorylated graphene oxide	138

Data Generation, DP Training and Validation

A schematic of the overall data generation, DP training and validation is shown in Figure 6 of the main text. The initial data set was generated to target essential aspects of the material: (1) conduction of protons, (2) rotation of hydroxyl groups, and (3) stability of

hydroxyl groups during proton conduction. *NVT* DFT-MD simulations (25 ps with 0.25 fs time steps) were run at high temperatures (T = 800 K and T = 1000 K) for the c24C system to sample proton hopping events. Hydroxyl group bond rotation configurations were sampled by randomly perturbing the bond angle (θ) made between each hydroxyl group and the axis perpendicular to the surface of relaxed u24C graphanol along the center of its corresponding O atom. Ten such perturbed structures were used as starting configurations to run *NVT* DFT-MD at T = 1000 K for 5 ps with 0.5 fs time steps. The third set of initial training configurations was generated by sampling random c24C structures from a DFT-MD simulation, finding the CEC in each of them and then moving the H_2O group towards and away from the plane of c24C. Single point energies were computed for each of these structures and added to the database. An ensemble of four DPs was generated using the active learning scheme as discussed above. The DP with the lowest loss function value was first used to validate the quality in the prediction of the phonon densities compared to DFT. This DP was then used to compute essential bond energetics/dynamics such as energy of the C−H bond, H_2O formation energy, and the hydroxyl group bond rotation energy, which were validated with DFT. This procedure determines the quality of the "best" DP for that given active learning iteration.

Another key part to the active learning process is the exploration step (MD exploration in Figure 6), which involves sampling of the configuration space and transferring these configurations to the labeling step. The error indicator from the exploration step was used as a metric to test the quality of DPs for that iteration. A successful iteration was one where the "best" DP passed the phonon test, bond energy tests, and achieved a low error (ϵ) from the MD exploration. An unsuccessful iteration resulted in another training iteration where configurations with maximal force deviations from the exploration step that fell within set error bounds were then relabeled using single-point DFT calculations and were added to the training data set.

CEC Trace-back Study

Determining the list of O atoms that form a Grotthuss chain (GC) at each time instant is computationally challenging. We took a heuristic approach to demonstrate the existence of a GC by measuring the time it takes for a proton traverse a set of O atoms and to re-trace its path back to its initial position. Data were collected from a NVT DP-MD simulation of a c24C cell, at $T = 800$ K, for 125 ps (0.25 fs time steps). We recorded the amount of time a CEC at time t (CEC_t) takes to follow a sequence of O atoms and then return along the same path back to CEC_t. Trace-back sequences must have more than three unique O atoms, including CEC_t. For example, A-B-C-D-C-B-A is a possible sequence that consists of four unique O atoms. We consider instances of two distinct O atoms as proton rattling motion and ignore these configurations. However, these rattling events were not ignored if they were part of a longer trace-back sequence. For example, A-B-C-D-C-D-C-B-A is an acceptable sequence with four unique O atoms even though there a C-D rattling event appears in it. We considered multiple trace-back sequences and estimated their trace-back times. We ensured that no two trace-back sequences overlapped at a given time, to avoiding over-counting. The time-distribution histogram obtained from this analysis is plotted in Figure 5, which contains all trace-back sequences (of various lengths). A log transform of this plot (Figure S9a). exhibits a normal distribution. This was verified using the QQ plot and the D'Agostino and Pearson's statistical tests.[21,22] According to our analysis, the mean proton trace-back time is 0.24 ps. A chain-length resolved histogram is plotted in Figure S9b-d. Trace-back sequences involving three unique O atoms dominate the distribution, whereas sequences of increasing length appear less and less frequently. This finding suggests that it is unlikely that a GC much longer than 6 survives for sufficiently long times for trace-back to occur, or that GCs much longer than 6 are unlikely to occur. Our analysis cannot distinguish between these two possibilities.

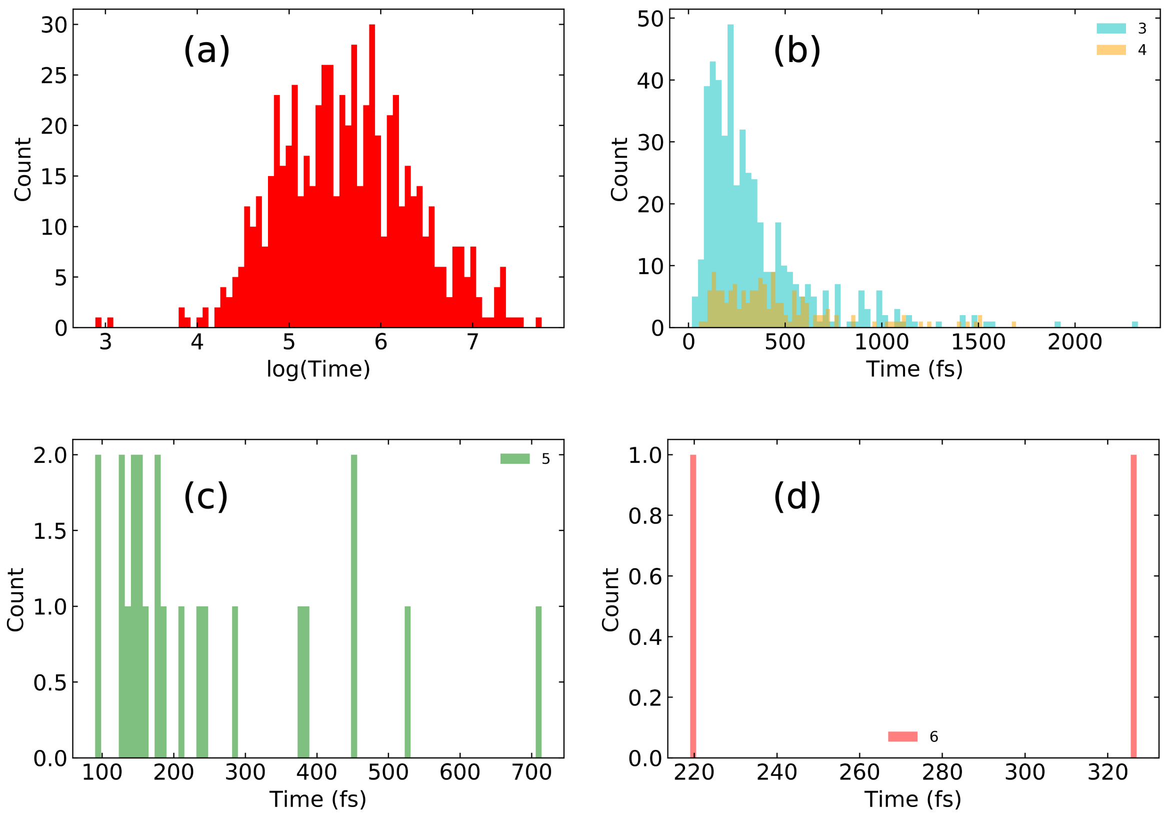

Figure S9: (a) Log transform of CEC trace-back time distribution histogram. (b) Component-wise histogram plot of Figure (a). Each color indicates the number of unique O atoms that constitute a trace-back sequence. (c) Histogram plot of CEC trace-back time distribution that contains a five unique O atom sequence. (d) Histogram plot of CEC trace-back time distribution that contains a six unique O atom sequence.

Lattice Monte Carlo Model

We developed an undirected graph model for charged graphanol (c24C) to perform lattice Monte Carlo (LMC) simulations. This model contain nodes that represent H atom sites and undirected edges that connect the nodes, as shown in Figure S10. A set of six nodes correspond to the neighbors of an O atom and the edge nodes are connected to their corresponding periodic images. We provide the pseudo code for simulating hydroxyl group rotations and proton hops in Algorithm 1. The graph was initially populated by adding H atoms in such a way that only one O atom (hexagon set) contains two occupied sites (the charged O atoms), while all others contain just one site. Each simulation involved for 10^6 moves, from which estimated the overall PT barrier, E_A. Prior to the first move, we set the OH rotation Boltzmann factor ($k_{\mathrm{rot}} = e^{\frac{-E_{\mathrm{rot}}}{RT}}$) and proton hop Boltzmann factor ($k_{\mathrm{hop}} = e^{\frac{-E_{\mathrm{hop}}}{RT}}$) using the initial set of E_{rot} and E_{hop} values. For each move, we randomly select an O atom, o, (shown as a hexagon surrounding a green O_i in Figure S10) and we compute the total number of H neighbors using $neighbors\_H()$. If the O atom contains only one H atom, an OH rotation move is attempted, otherwise a proton hop move is attempted. Attempts at rotation or proton hopping are successful only if a set of conditions are met. A rotation can occur only if o is *not* the CEC, otherwise a proton hop is attempted. A successful rotation is performed only when the site of the rotation has no adjacent neighbors that are occupied *and* provided a random number ζ from a uniform distribution $U(0, 1)$ is lower than k_{rot}. When o corresponds to the CEC, we identify a set of potential hopping sites. If a hopping site has no occupied neighbors *and* a random number ζ from $U(0, 1)$ is lower than k_{hop}, then a hopping event occurs. The Cartesian coordinates of the CEC determined at each *move* were used to calculate the MSD of the CEC, to finally estimate E_A. We assume a time step of 0.25 fs and increment the clock after each attempted move.

Algorithm 1 Lattice Monte Carlo in c24C graphanol

```
Randomly populate nodes
k_rot = e^(-E_rot/RT)                                   ▷ OH rotation Boltzmann factor
k_hop = e^(-E_hop/RT)                                   ▷ Proton hop Boltzmann factor
while moves ≤ 10^6 do
    o ← pick random O atom
    if len(neighbors_H(o)) == 1 then                    ▷ Rotation attempt
        h ← neighbors_H(o)
        if len(rot_sites(h)) == 0 then
            reject rotation
        else
            if len(rot_sites(h)) == 2 then
                rot_pick ← random(rot_sites(h))
            else                                        ▷ len(rot_sites(h)) == 1
                rot_pick ← rot_sites(h)
            end if
            if len(neighbors_H(rot_pick)["occupied"] == True) > 0 then
                reject rotation
            else
                ζ ← U(0, 1)
                if ζ < k_rot then
                    accept rotation
                else
                    reject rotation
                end if
            end if
        end if
    else                            ▷ Proton hop attempt, len(neighbors_H(o)) == 2
        h ← random(neighbors_H(o))
        hop_pick ← hop_site(h)                          ▷ Can only be max. of one hop site
        if hop_pick == None then
            reject hop
        else if len(neighbors_H(hop_pick)["occupied"] == True) > 0 then
            reject hop
        else
            ζ ← U(0, 1)
            if ζ < k_hop then
                accept hop
            else
                reject hop
            end if
        end if
    end if
end while
```

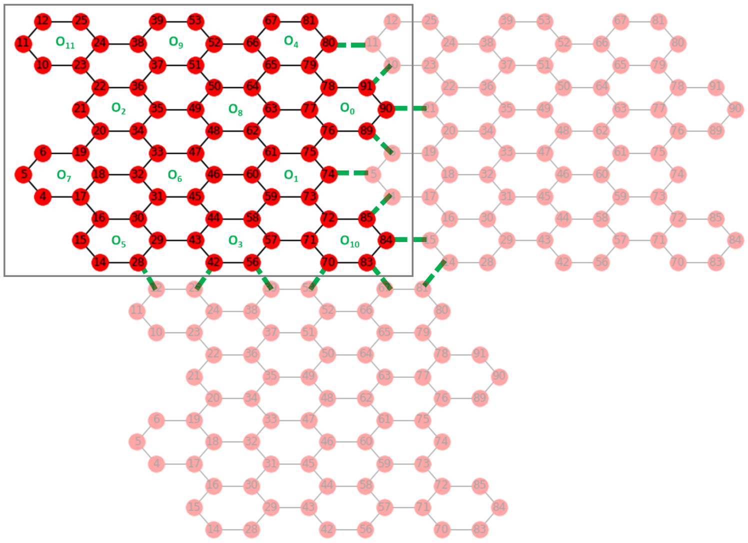

Figure S10: Graph model to perform lattice Monte Carlo simulations on 2-D single-sided graphanol. The red nodes depict all the possible H atom sites and edges between the nodes decide where these H atoms can move. The grey box defines the simulation cell and the lightly shaded nodes and edges are representative periodic images along two directions.

References

(1) Blöchl, P. E. Projector Augmented-Wave Method. *Phys. Rev. B* **1994**, *50*, 17953.

(2) Perdew, J. P.; Burke, K.; Ernzerhof, M. Generalized Gradient Approximation Made Simple. *Phys. Rev. Lett.* **1996**, *77*, 3865.

(3) Zhang, Y.; Yang, W. Comment on “Generalized Gradient Approximation Made Simple”. *Phys. Rev. Lett.* **1998**, *80*, 890.

(4) Bagusetty, A.; Choudhury, P.; Saidi, W. A.; Derksen, B.; Gatto, E.; Johnson, J. K. Facile Anhydrous Proton Transport on Hydroxyl Functionalized Graphane. *Phys. Rev. Lett.* **2017**, *118*, 186101.

(5) Evans, D. J.; Holian, B. L. The Nose–Hoover Thermostat. *J. Chem. Phys.* **1985**, *83*, 4069–4074.

(6) Li, C.; Swanson, J. M. Understanding and Tracking the Excess Proton in Ab Initio Simulations; Insights from IR spectra. *J. Phys. Chem. B* **2020**, *124*, 5696–5708.

(7) Knight, C.; Lindberg, G. E.; Voth, G. A. Multiscale Reactive Molecular Dynamics. *J. Chem. Phys.* **2012**, *137*, 22A525.

(8) Wu, X.; Thiel, W.; Pezeshki, S.; Lin, H. Specific Reaction Path Hamiltonian for Proton Transfer in Water: Reparameterized Semiempirical Models. *J. Chem. Theory Comput.* **2013**, *9*, 2672–2686.

(9) Cao, B.-Y.; Dong, R.-Y. Molecular Dynamics Calculation of Rotational Diffusion coefficient of a carbon nanotube in fluid. *J. Chem. Phys.* **2014**, *140*, 034703.

(10) Fu, Y.-Z.; Manthiram, A. Nafion–Imidazole– H3PO4 Composite Membranes for Proton Exchange Membrane Fuel Cells. *J. Electrochem. Soc.* **2006**, *154*, B8.

(11) Lei, M.; Wang, Y.; Zhang, F.; Huang, C.; Xu, X.; Zhang, R.; Fan, D. Anhydrous Proton Conducting Composite Membranes Containing Nafion and Triazole Modified POSS. *Electrochim. Acta* **2014**, *149*, 206–211.

(12) Sen, U.; Bozkurt, A.; Ata, A. Nafion/poly(1-vinyl-1,2,4-triazole) Blends as Proton Conducting Membranes for Polymer Electrolyte Membrane Fuel Cells. *J. Power Sources* **2010**, *195*, 7720–7726.

(13) Yuan, S.; Guo, X.; Aili, D.; Pan, C.; Li, Q.; Fang, J. Poly(imide benzimidazole)s for High Temperature Polymer Electrolyte Membrane Fuel Cells. *J. Membr. Sci.* **2014**, *454*, 351–358.

(14) Mader, J. A.; Benicewicz, B. C. Sulfonated Polybenzimidazoles for High Temperature PEM Fuel Cells. *Macromolecules* **2010**, *43*, 6706–6715.

(15) Ghosh, S.; Maity, S.; Jana, T. Polybenzimidazole/silica Nanocomposites: Organic-inorganic Hybrid Membranes for PEM Fuel Cell. *J. Mater. Chem.* **2011**, *21*, 14897–14906.

(16) Barati, S.; Abdollahi, M.; Khoshandam, B.; Mehdipourghazi, M. Highly Proton Conductive Porous Membranes Based on Polybenzimidazole/ Lignin Blends for High Temperatures Proton Exchange Membranes: Preparation, Characterization and Morphology-Proton Conductivity Relationship. *Int. J. Hydrog. Energy* **2018**, *43*, 19681–19690.

(17) Chen, J.-C.; Wu, J.-A.; Lee, C.-Y.; Tsai, M.-C.; Chen, K.-H. Novel Polyimides Containing Benzimidazole for Temperature Proton Exchange Membrane Fuel. *J. Membr. Sci.* **2015**, *483*, 144–154.

(18) Zhang, J.; Lu, S.; Zhu, H.; Chen, K.; Xiang, Y.; Liu, J.; Forsyth, M.; Jiang, S. P. Amino-functionalized Mesoporous Silica Based Polyethersulfone–polyvinylpyrrolidone Composite Membranes for Elevated Temperature Proton Exchange Membrane Fuel Cells. *RSC Adv.* **2016**, *6*, 86575–86585.

(19) Bai, H.; Wang, H.; Zhang, J.; Zhang, J.; Lu, S.; Xiang, Y. High Temperature Polymer Electrolyte Membrane Achieved by Grafting poly(1-vinylimidazole) on polysulfone for Fuel Cells Application. *J. Membr. Sci.* **2019**, *592*, 117395.

(20) Bai, H.; Li, Y.; Zhang, H.; Chen, H.; Wu, W.; Wang, J.; Liu, J. Anhydrous Proton Exchange Membranes Comprising of Chitosan and Phosphorylated Graphene Oxide for Elevated Temperature Fuel Cells. *J. Membr. Sci.* **2015**, *495*, 48–60.

(21) D'Agostino, R.; Pearson, E. S. Tests for Departure from Normality. Empirical Results for the Distributions of b^2 and $\sqrt{b}$. *Biometrika* **1973**, *60*, 613–622.

(22) D'Agostino, R. An Omnibus Test of Normality for Moderate and Large Sample Sizes. *Biometrika* **1971**, *58*, 1–348.
